# Comparative Proteomic Analysis Reveals That TaCAD-A1 Enhances Resistance of Wheat to Powdery Mildew (*Blumeria graminis* f. sp. *tritici*)

**DOI:** 10.3390/life16060872

**Published:** 2026-05-22

**Authors:** NiNa Sun, Wei Liu, WeiHua Xu, LinZhi Li, TangYu Yuan, Lu Chen

**Affiliations:** 1 Yantai Academy of Agricultural Sciences, Yantai 265500, China; sun200436@163.com (N.S.); liuweisdau@163.com (W.L.); xuweihuayt@163.com (W.X.); linzhili2002@163.com (L.L.); 2 State Key Laboratory of Agricultural Products Safety, Ningbo University, Ningbo 315211, China; 3 Key Laboratory of Biotechnology in Plant Protection of MARA, Ningbo University, Ningbo 315211, China; 4 Zhejiang Key Laboratory of Green Plant Protection, Institute of Plant Virology, Ningbo University, Ningbo 315211, China

**Keywords:** wheat, *Blumeria graminis* f. sp. *tritici*, tandem mass tag (TMT)-based proteomics analysis, defense-related proteins, TaCAD-A1

## Abstract

Powdery mildew in wheat, caused by the biotrophic fungus *Blumeria graminis* f. sp. *tritici* (*Bgt*), is a major threat to global wheat production, yet the molecular mechanisms underlying differential cultivar resistance remain largely unresolved. In this study, tandem mass tag (TMT)-based quantitative proteomics was employed to investigate protein dynamics in resistant (Yannong37) and susceptible (Yannong1766) wheat cultivars at 0 and 24 h following *Bgt* inoculation. A total of 276 proteins exhibited significant changes in abundance after infection, with enrichment in cell wall and plasmodesmata-associated proteins. Comparative analysis further identified 456 differentially expressed proteins between the two cultivars at 24 h post-inoculation. Protein–protein interaction network analysis indicated that proteins involved in secondary metabolism and immune responses form coordinated regulatory networks contributing to disease resistance. RT–qPCR validation supported the reliability of the proteomic data. Notably, *TaCAD-A1* displayed higher transcript abundance in the resistant cultivar and was associated with reduced fungal biomass accumulation. Silencing of *TaCAD-A1* resulted in decreased expression of multiple defense-related genes. Collectively, these findings suggest that *TaCAD-A1* may positively contribute to wheat resistance against *Bgt* infection and may be associated with defense responses and monolignol biosynthesis-related processes.

## 1. Introduction

Wheat (*Triticum aestivum* L.) is a major staple crop worldwide. The fungal pathogen *Blumeria graminis* f. sp. *tritici* (*Bgt*), the etiological agent of wheat powdery mildew, poses a critical threat to global wheat production systems [1,2,3]. The strategic deployment of host resistance represents the cornerstone of integrated powdery mildew management, demonstrating 60–85% disease suppression efficacy in multi-location field trials [4,5,6].

The mechanisms of plant resistance to powdery mildew are primarily manifested through the reinforcement of the cell wall, including papillae, and the occurrence of a hypersensitive response (HR). Relevant studies at the molecular level have revealed the importance of genes encoding cellulose and callose in the formation of effective papillae in barley, highlighting the importance of glucans in the development of barley papillae [7,8]. Existing models of plant immunity propose that nucleotide-binding domain and leucine-rich repeat (NLR) proteins trigger potent broad-spectrum immune reactions by monitoring the activity of pathogen-secreted molecules known as effectors [9]. This process triggers HR, a form of programmed cell death (PCD) localized at the site of infection to limit pathogen spread [10,11]. To date, numerous wheat powdery mildew resistance genes, such as *Pm2* [12], *Pm3* [13], *Pm8Pm17* [14], *Pm21* [15,16] and *Pm36* [17], have been identified and cloned in wheat. The majority of functionally characterized disease resistance genes (*R*-genes) in cereal crops belong to the NLR superfamily and these genes have the potential capacity to increase resistance [15]. In addition to NLRs, Wang et al. reported that the glutathione S-transferase TaGST6 interacts with the TaCBSX3, which is involved in resistance to *Bgt* but not *Pst* [18]. Another study indicated that the use of the transcription factor TaNAC1 and its regulators is a strategy for improving wheat resistance to fungal pathogens [19]. However, prolonged survival challenges and environmental pressures have caused the evolution of new virulent pathogenic strains, resulting in resistance genes of wheat becoming less effective against powdery mildew [20]. Therefore, identifying new sources of broad-spectrum resistance to control diseases is necessary.

Proteomics is the study of proteomes and focuses on the overall composition and regulatory activities of proteins, mainly the expression and functional patterns of proteins. With the development of omics technologies, proteomic approaches have been extensively utilized in the study of plant–pathogen interactions to mine new sources of disease resistance [21,22,23]. In early studies, the most commonly used methods were a combination of two-dimensional electrophoresis (2-DE) and MALDI-TOF MS (Matrix-Assisted Laser Desorption/Ionization Time-of-Flight Mass Spectrometry) [24]. Wang et al. compared the leaf proteins of Jingdong8 (JD8) and JD8 with a single *Pm* resistance gene, *Pm30* (*JD8-Pm30*), and observed differentially expressed proteins involved in signal transduction, disease and defense. Some results revealed the relevance of primary plant metabolism and defense responses during compatible interactions [25]. Proteome surveys have investigated resistance proteins associated with *Bgt* inoculation in the wheat lines L699 (with *Pm40*), Neimai836 (with *Pm21*), and Chuannong26, which are involved in physiological and cellular processes related to wheat resistance to *Bgt* [26]. Proteomic analysis utilizing tandem mass tags (TMTs) has been demonstrated to be an effective approach for identifying differentially expressed proteins in wheat in response to *Fusarium pseudograminearum* infection [27]. These findings demonstrate that proteomics analysis is a highly effective approach to uncover the deferentially expressed proteins between resistant and susceptible wheat cultivars in response to *Bgt*. Nevertheless, no in-depth studies employing TMT-based proteomic analysis have been conducted to compare the interactions between wheat and *Bgt* in two wheat varieties with varying degrees of resistance to powdery mildew.

On the basis of the current understanding, we decided to utilize the recent advancements in TMT quantitative proteomics technology to analyze the global proteome in both resistant and susceptible wheat varieties during *Bgt* inoculation. We explore the possible roles of these proteins in the early defense response phase based on their functions. Our objectives were to not only enhance our comprehension of the interaction between wheat and *Bgt* but also to contribute to the creation of more efficient methods to manage this destructive pathogen. We believe this study will introduce novel perspectives for innovating wheat powdery mildew-resistant germplasm resources and promoting the genetic improvement and breeding of new wheat varieties.

## 2. Materials and Methods

### 2.1. The Plant Materials, Inoculation and Cytological Observation

Yannong37 and Yannong1766 were used in this research. Yannong37 is resistant to most powdery mildews isolated in China; Yannong1766 is highly vulnerable to powdery mildew, with no effective resistance genes present. The plants were grown in pots with a diameter of 8–10 cm in a greenhouse growth chamber, maintained at 18 °C and under a 12 h light/12 h dark photoperiod. These pots were divided into two groups, one treated with mock inoculation and the other with *Bgt* inoculation. Approximately 10 days post-sowing, the seedlings were artificially inoculated with *Bgt* isolate E09. The pots were shielded with translucent and aerated covers to avoid fungal cross-contamination. These seedling samples were harvested at 0 and 24 hpi with liquid nitrogen and stored at −80 °C.

Samples from the mock-inoculated group and *Bgt*-inoculated group were collected from three biological replicates at two time points (0 and 24 hpi). The leaf tissues were cut into 2–4 cm fragments and placed in 2 mL EP tubes. The samples were decolorized using an AA solution (a mixture of ethanol and glacial acetic acid in a 1:1 volume ratio) at 70 °C for 20 min. The samples were then stained with a Coomassie Brilliant Blue staining solution (a mixture of 0.15% trichloroacetic acid aqueous solution and 0.6% Coomassie Brilliant Blue R-250 methanol solution in a 1:1 ratio) for 4 h for cytological observation. The infection structure of *Bgt* was observed under a microscope (Olympus, Tokyo, Japan) [18].

### 2.2. Total Protein Extraction and Protein Trypsin Digestion

For the proteomics experiments, total proteins were extracted from wheat leaves using the TCA/acetone precipitation method [21]. Leaf tissues were homogenized in liquid nitrogen and resuspended in lysis buffer (40 mM Tris-HCl pH 8.5, 2 M thiourea, 7 M urea, 10 mM DTT, 4% SDS, 2 mM EDTA, 1 mM PMSF). After centrifugation (15,000 *g*, 10 min, 4 °C), supernatants were precipitated with 4 volumes of acetone overnight at −20 °C. The pellet was washed thrice with acetone/ethanol (1:1), air-dried, and solubilized in 8 M urea/100 mM TEAB (pH 8.0). Protein concentration was quantified by BCA assay.

### 2.3. LC–MS/MS Analysis and Proteomic Data Analysis

Peptides were solubilized in 0.1% formic acid (solvent A) and analyzed via Vanquish Neo UHPLC (Thermo Fisher Scientific, Waltham, MA, USA) coupled with Orbitrap Eclipse mass spectrometer (Thermo Fisher Scientific, Waltham, MA, USA). A 1 µL aliquot was injected onto a trapping column (Acclaim PepMap 100, 75 µm × 2 cm, C18) (Thermo Fisher Scientific, Waltham, MA, USA) at 10 µL/min for 3 min. Separation was achieved on an analytical column (Acclaim PepMap 100, 75 µm × 25 cm, C18) (Thermo Fisher Scientific, Waltham, MA, USA) using a gradient from 2.5% to 23% solvent B (80% acetonitrile/0.1% FA) over 100 min, followed by 23–32% in 20 min and 32–90% in 19 min (total 139 min). MS data were acquired in DDA mode with 2 s cycle time: full scans (300–2000 *m*/*z*, 120 k resolution, AGC 400 k, 50 ms IT) followed by HCD-MS/MS (1.6 *m*/*z* window, 30% NCE, AGC 50 k, 54 ms IT) at 300 nL/min (50 °C). This workflow enables sensitive analysis of complex peptide mixtures.

### 2.4. Statistical and Bioinformatic Analysis of Proteomic Data

Differentially expressed proteins (DEPs) were identified based on a fold change >1.5 or <0.67 and *p* < 0.05. Protein quantification was performed using three biological replicates for each treatment, and statistical analyses were conducted using Student’s *t*-test. Prior to statistical analysis, protein abundance values were normalized to minimize technical variation. Proteins showing significant abundance changes between treatments were considered DEPs and subjected to subsequent bioinformatic analyses. Detailed information for all identified DEPs is provided in Supplementary Table S3.

Functional annotation of the identified proteins was performed using the UniProt-GOA database (European Bioinformatics Institute, Cambridge, UK), accessed on 20 May 2021). GO enrichment and KEGG pathway analyses of DEPs were conducted using the online KOBAS platform. The expression levels of DEPs enriched in GO terms and KEGG pathways were transformed into log2 fold changes with *p* < 0.05. Protein–protein interaction (PPI) networks were constructed using STRING software (version 11.5; https://string-db.org/) and visualized using Cytoscape software (version 3.8.0).

### 2.5. Total RNA Extraction and RT–qPCR

Total RNA was extracted from non-inoculated control and *Bgt*-inoculated wheat seedlings using the HiPure Plant RNA Mini Kit (Magen, Guangzhou, China), followed by first-strand cDNA synthesis with oligo (dT) primers via the TOYOBO First Strand cDNA Synthesis Kit (Toyobo, Osaka, Japan). Quantitative PCR analysis employing SYBR Green chemistry (Vazyme, Nanjing, China) on Applied Biosystems QuantStudio 6 Flex system was performed (Applied Biosystems, Foster City, CA, USA) using gene-specific primers, with wheat actin (*TaACT*) as internal reference. Primers were designed using the NCBI Primer-BLAST tool based on the target gene sequences. The primer sequences used in this study are listed in Supplementary Table S5. PCR amplification was performed under the following conditions: initial denaturation at 95 °C for 3 min, followed by 35 cycles of 95 °C for 15 s, annealing at the primer-specific temperature (57–60 °C) for 15 s, and 72 °C for 20 s, with a final extension at 72 °C for 5 min. Relative expression levels were calculated by the 2^−ΔΔct^ method, with three biological replicates and technical triplicates.

### 2.6. BSMV-Mediated Gene Silencing

The BSMV-based VIGS assay was performed as previously reported [18]. Briefly, to generate the γ:*TaCAD-A1* constructs, 300 bp antisense fragments of γ:*TaCAD-A1* were predicted using the website https://vigs.solgenomics.net/ and subcloned individually into the pBSMVγ vector. The plasmids pBSMVα, pBSMVβ, pBSMVγ, pBSMVγ:*TaCAD-A1*, and pBSMVγ:*TaPDS* were individually linearized with specific restriction enzymes. Subsequently, RNA transcripts were generated from these linearized plasmids using the mMessage mMachine T7 in vitro transcription kit according to the manufacturer’s instructions (Ambion, Austin, TX, USA). Mixtures of pBSMVα and pBSMVβ together with pBSMVγ, pBSMVγ:*TaCAD-A1* and pBSMVγ:*TaPDS* were inoculated onto the second leaf of wheat seedling leaves. At 10 dpi with BSMV, the leaves of each wheat seedling were inoculated with *Bgt*, and the samples were harvested at 24 hpi for silencing efficiency determination and histological observation.

## 3. Results

### 3.1. Powdery Mildew Infection Increased Protein Abundance in Resistant Wheat Cultivars

**Figure 1:**
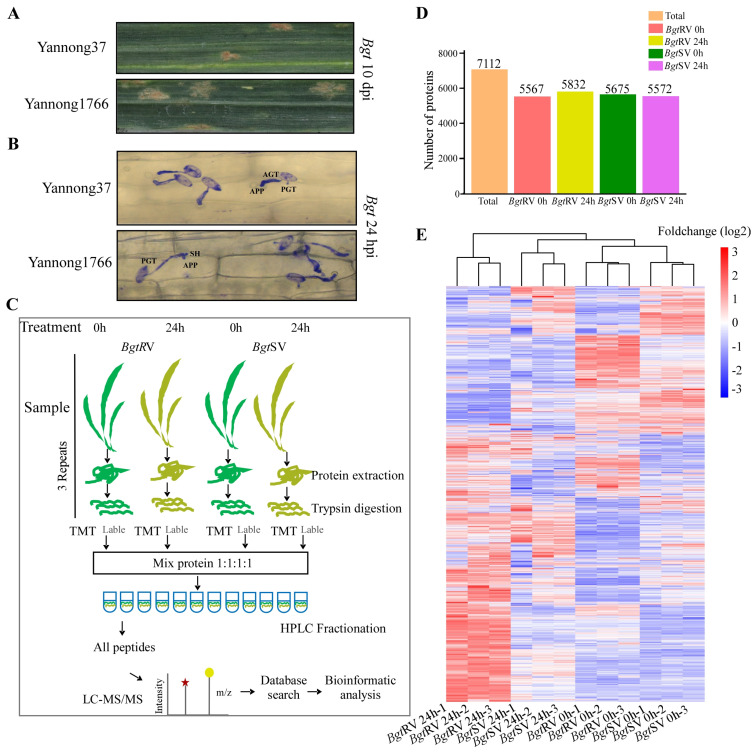
Large*−*scale proteomics analyses of wheat cultivars Yannong37 and Yannong1766 inoculated with *Bgt*. (**A**) The wheat variety resistant to powdery mildew Yannong37 (*Bgt*RV) and the wheat variety susceptible to powdery mildew Yannong1766 (*Bgt*SV) infected with *Bgt* at 10 dpi. (**B**) The growth of *Bgt* in wheat leaves was observed via microscopy. Wheat leaves inoculated with *Bgt* were sampled at 24 hpi. Scale bars = 20 μm; PGT, primary germ tube; AGT, appressorial germ tube; APP, hooked apical appressorium; SH, secondary hyphae. (**C**) The experimental workflow for the proteomics analysis is shown. Red asterisks (★) and yellow circles (●) mark peptide peaks from the control and treatment groups, respectively. (**D**) Bar chart displaying the overlaps of DEPs during *Bgt* inoculating. (**E**) Hierarchical clustering analysis of DEPs in Yannong37 and Yannong1766 during the 24 hpi with *Bgt* inoculated. The increased or decreased proteins are marked in red or blue, respectively.

Large-scale proteomic analysis was performed using the *Bgt*-resistant wheat cultivar Yannong37 (*Bgt*RV) and the *Bgt*-susceptible cultivar Yannong1766 (*Bgt*SV) at 0 and 24 h post-inoculation (hpi). Phenotypic and histological analyses indicated that Yannong37 is highly resistant to *Bgt*. At 10 days post-inoculation (dpi), Yannong1766 developed severe disease symptoms, whereas Yannong37 showed no obvious symptoms (Figure 1A). Histological observations at 24 hpi further showed that, compared with Yannong1766, Yannong37 significantly restricted fungal growth, as evidenced by shorter hyphae and a smaller infection area (Figure 1B). After total protein extraction, trypsin digestion and labeling with isobaric tags, the samples were subjected to LC–MS/MS for analysis and identification. Three biological replicates were included at each time point. An overview of the proteomic identification assay workflow is presented in Figure 1C. A total of 7112 proteins were quantified, and 5567, 5832, 5675 and 5572 proteins were quantified in inoculated *Bgt*RV wheat seedlings at 0 hpi and 24 hpi (*Bgt*RV 0 h and *Bgt*RV 24 h) and inoculated *Bgt*SV wheat seedlings at 0 h and 24 h hpi, respectively (Figure 1D). To investigate the accumulation patterns of the quantified proteins in *Bgt*RV and *Bgt*SV during *Bgt* inoculation, we performed hierarchical clustering analysis using the intensity ratios normalized to the protein abundance. The protein abundance did not significantly change in *Bgt*SV 0 h and *Bgt*SV 24 h wheat seedlings but significantly increased 24 hpi with *Bgt* in *Bgt*RV wheat seedlings (Figure 1E and Supplementary Table S1).

### 3.2. The Abundance of Proteins Enriched in the Cell Wall and Plasmodesmata Significantly Increased in the Resistant Wheat Variety

**Figure 2:**
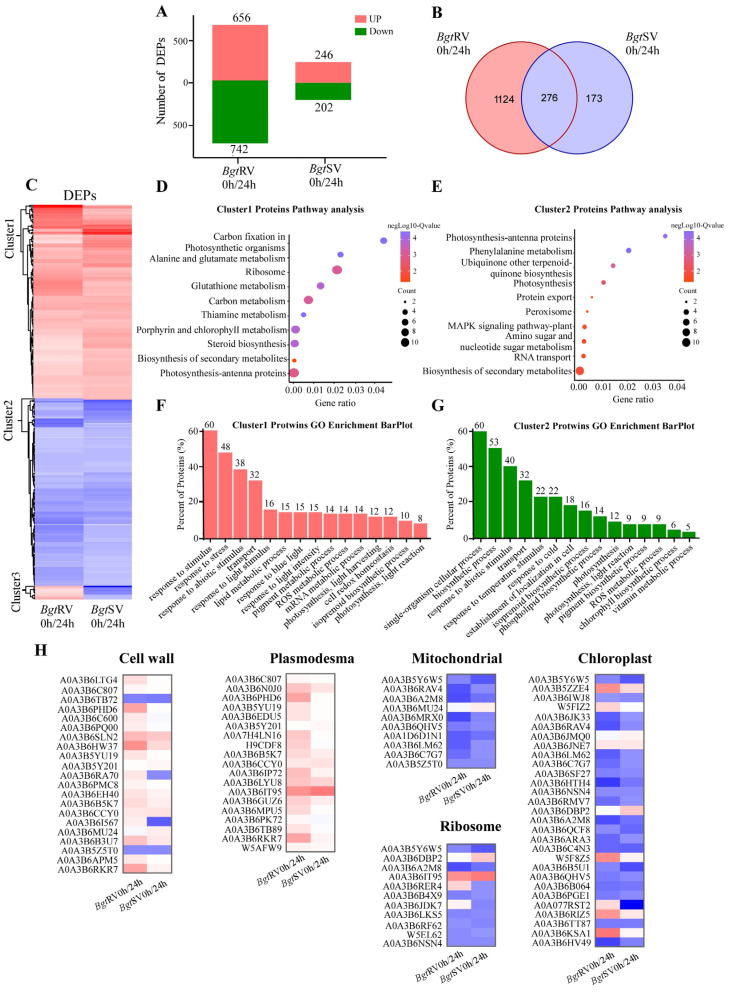
Overview of DEPs in *Bgt*RV 0 h/24 h and *Bgt*SV 0 h/24 h. (**A**) Bar chart displaying number of DEPs in *Bgt*RV 0 h/24 h and *Bgt*SV 0 h/24 h; (**B**) Venn diagrams are utilized to illustrate the intersections between the *Bgt*RV 0 h/24 h and *Bgt*SV 0 h/24 h. (**C**) Hierarchical clustering analysis of DEPs. The two columns correspond to distinct treatments of *Bgt*RV 0 h/24 h and *Bgt*SV 0 h/24 h; (**D**) KEGG pathway enrichment analysis of *Bgt*RV 0 h/24 h and *Bgt*SV 0 h/24 h differentially upregulated proteins; (**E**) downregulated proteins; (**F**) GO enrichment analysis of *Bgt*RV 0 h/24 h and *Bgt*SV 0 h/24 h differentially upregulated proteins (**G**) downregulated proteins in the biological processes; (**H**) heatmap analysis of DEPs in *Bgt*RV 0 h/24 h and *Bgt*SV 0 h/24 h in different organelles. The squares colored by fold change. Red squares indicate upregulation and blue squares indicate downregulation.

Protein abundance is influenced by multiple biological and technical factors. Differentially expressed proteins (DEPs) were identified using a stringent threshold of fold change >1.5 or <0.67 with *p* < 0.05 based on three biological replicates. Detailed information on the identified DEPs is provided in Supplementary Table S3. At 24 h post-inoculation (hpi), a total of 448 DEPs were identified in susceptible wheat seedlings infected with *Bgt*, including 246 significantly upregulated proteins and 202 significantly downregulated proteins. In comparison, a greater number of DEPs were identified in resistant wheat seedlings following *Bgt* infection. A total of 1398 DEPs were identified between *Bgt*RV 0 h and *Bgt*RV 24 h, among which 656 proteins were significantly upregulated and 742 proteins were significantly downregulated (Figure 2A). To identify the proteins regulated by *Bgt* inoculation, we screened 276 common proteins between *Bgt*RV 0 h and *Bgt*RV 24 h and between *Bgt*SV 0 h and *Bgt*SV 24 h (Figure 2B). Hierarchical cluster analysis was executed with 276 DEPs to explore the molecular patterns associated with *Bgt* inoculation; most of these patterns tended to change in the same direction in Clusters 1 and 2, whereas the changes were greater in *Bgt*RV 0 h than in *Bgt*RV 24 h (Figure 2C). To better understand these results, we performed a Kyoto Encyclopedia of Genes and Genomes (KEGG) pathway functional enrichment analysis. Photosynthesis-antenna proteins and biosynthesis of secondary metabolites were enriched in both Cluster 1 and Cluster 2 (Figure 2D,E). To investigate the DEP-associated pathways affected upon *Bgt* inoculation, a Gene Ontology (GO) enrichment analysis was performed on the DEPs in Clusters 1 and 2. Fifteen biological process (BP) terms, including lipid metabolic process, ROS metabolic process, photosynthesis and isoprenoid biosynthetic process, were significantly enriched in Cluster 1, and the ratios of the DEPs in Cluster 2 involved in transport, response to temperature stimulus, establishment of localization in cells and vitamin metabolic process were greater, suggesting that these proteins play important roles in *Bgt* inoculated in wheat (Figure 2F,G). The abundance of proteins in different subcellular organelles of *Bgt*-inoculated wheat seedling leaves is known to be affected. *Bgt*RV 0 h vs. *Bgt*RV 24 h and *Bgt*SV 0 h vs. *Bgt*SV 24 h protein abundances in different subcellular organelles tended to change in the same direction. In this study, protein abundance was significantly increased within the plasmodesmata and the cell wall upon *Bgt* inoculation, whereas protein abundance was significantly decreased within ribosomes, mitochondria, and chloroplasts. Notably, compared with that in the susceptible variety, the abundance of proteins enriched in the cell wall and plasmodesmata was greater after powdery mildew infection in the resistant variety (Figure 2H) and Supplementary Table S2, which is possibly related to the change in the abundance of proteins associated with the biosynthesis of secondary metabolism.

### 3.3. The Abundance of Immune-Related Proteins Significantly Differed Between Resistant and Susceptible Wheat Cultivars upon Bgt Inoculation

**Figure 3:**
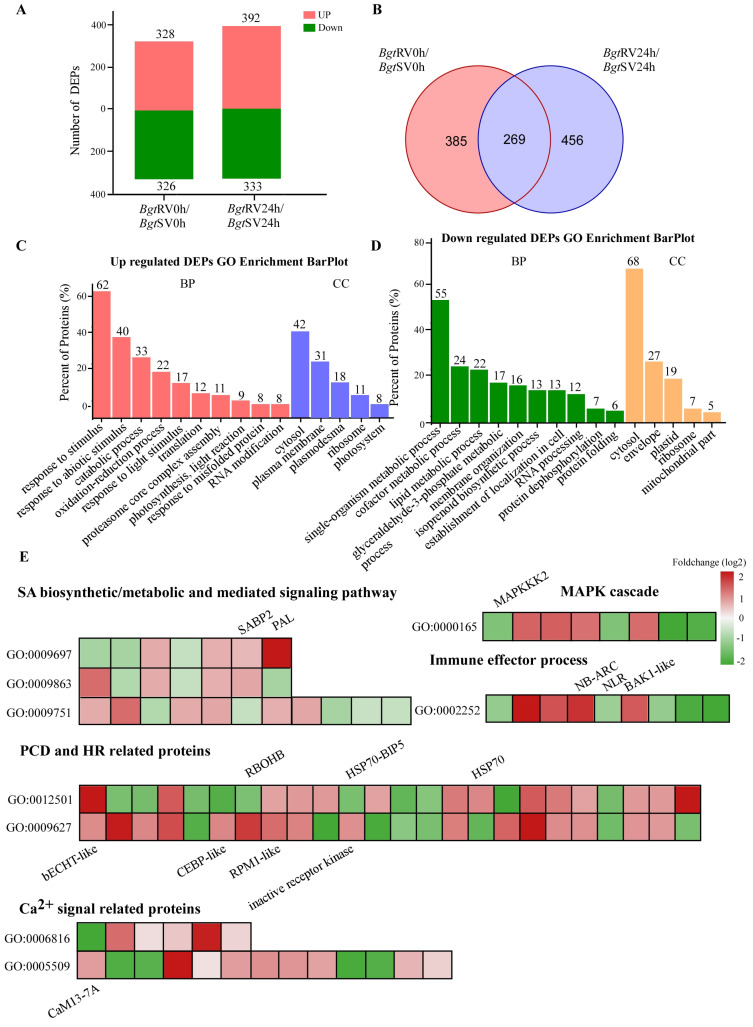
Overview of DEPs in *Bgt*RV 24 h/B*gt*SV 24 h. (**A**) Bar chart displaying number of DEPs in *Bgt*RV 0 h/*Bgt*SV 0 h and *Bgt*RV 24 h/*Bgt*SV 24 h; (**B**) Venn diagrams are utilized to illustrate the intersections between the *Bgt*RV 0 h/*Bgt*SV 0 h and *Bgt*RV 24 h/*Bgt*SV 24 h; (**C**) GO enrichment analysis of *Bgt*RV 24 h/*Bgt*SV 24 h differentially upregulated proteins (**D**) downregulated proteins in the biological processes (BP) and cellular components (CC); (**E**) heatmap of GO enriched terms of classic immune-related processes.

To better understand the molecular mechanism of resistance in *Bgt*RV, we systematically investigated the proteome dynamics of *Bgt*-inoculated *Bgt*RV wheat seedling leaves (Figure 3A). Compared with *Bgt*SV, 456 unique DEPs were identified in *Bgt*RV after *Bgt* was inoculated for 24 h (Figure 3B). To reveal the pathways regulated during *Bgt* inoculation of *Bgt*RV wheat seedling leaves, GO enrichment analysis of 456 unique DEPs was performed. We found that ten biological process (BP) terms and five cellular component (CC) terms were highly enriched in the upregulated and downregulated DEPs, respectively (Figure 3C,D). The abundance of immune-related proteins within wheat seedling leaves significantly changed following Bgt inoculation. We investigated the expression patterns of proteins associated with disease resistance pathways in *Bgt*RV wheat seedling leaves, including SA biosynthetic/metabolic and SA-mediated signaling pathways, the MAPK cascade, Ca^2+^ signal-related proteins, immune effector processes and PCD- and HR-related proteins. The abundances of salicylic acid-binding protein2-like (SABP2), phenylalanine ammonia-lyase (PAL), Calmodulin3-7A (Cam3-7A), NB-ARC domain-containing protein (NB-ARC), LRR receptor kinase BAK1-like (BAK1-like), basic endochitinase A-like (bECHT-like), chitin elicitor-binding protein-like (CEBP-like), disease resistance protein RPM1-like (RPM1-like) and heat shock cognate 70 kDa protein-like (HSP70) were significantly increased in BgtRV wheat seedling leaves, whereas the abundances of mitogen-activated protein kinase kinase kinase kinase 2 (MAPKKKK2), NLR family CARD domain-containing protein 3-like (NLR), respiratory burst oxidase homolog protein B-like (RBOHB) and heat shock 70 kDa protein BIP5-like (HSP70-BIP5) were significantly decreased in *Bgt*RV wheat seedling leaves (Figure 3E and Supplementary Table S4).

### 3.4. Biosynthesis of Secondary Metabolite Proteins Forms an Interaction Network with Immune-Related Proteins

**Figure 4:**
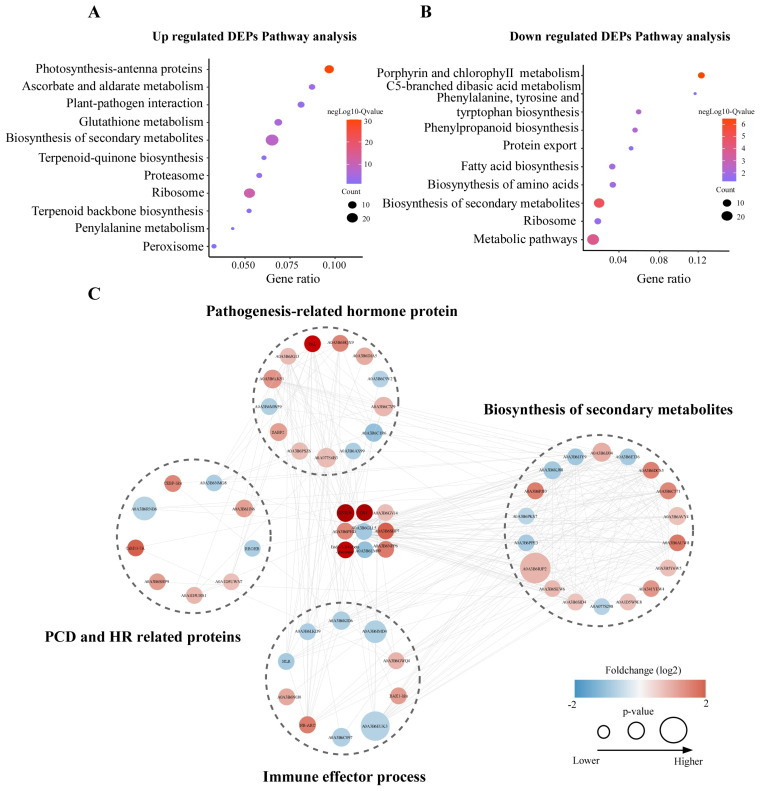
KEGG pathway and interaction network analyses of DEPs in *Bgt*RV 24 h/*Bgt*SV 24 h. (**A**) KEGG pathway enrichment analysis of *Bgt*RV 24 h/*Bgt*SV 24 h differentially upregulated proteins (**B**) downregulated proteins; (**C**) interaction networks for DEPs in *Bgt*RV 24 h/*Bgt*SV 24 h. The *Q*−value is a corrected *p*−value that ranges from 0 to 1, with lower values indicating greater significance. Circles depict the number of enriched genes, where larger circles signify a higher number of enriched genes.

KEGG analysis revealed that the upregulated DEPs were putatively involved in plant–pathogen interactions, glutathione metabolism, and the biosynthesis of secondary metabolites (Figure 4A), whereas the downregulated DEPs were putatively involved in metabolic pathways, porphyrin metabolism, chlorophyll metabolism and C5-branched dibasic acid metabolism pathways (Figure 4B). To model the dynamics of the immune-related proteins upon *Bgt* inoculation, we constructed a protein–protein interaction (PPI) network to identify functional modules. We identified five modules among the proteins that were highly interconnected, including pathogenesis-related hormone proteins, PCD- and HR-related proteins, immune effector processes and the biosynthesis of secondary metabolites (Figure 4C). Therefore, we propose that some proteins involved in the biosynthesis of secondary metabolites form complex interaction networks with classic immune-related proteins to participate in wheat resistance to powdery mildew infection.

### 3.5. Gene Expression Analysis of Selected DEP Genes in Interaction Network via qRT–PCR

**Figure 5:**
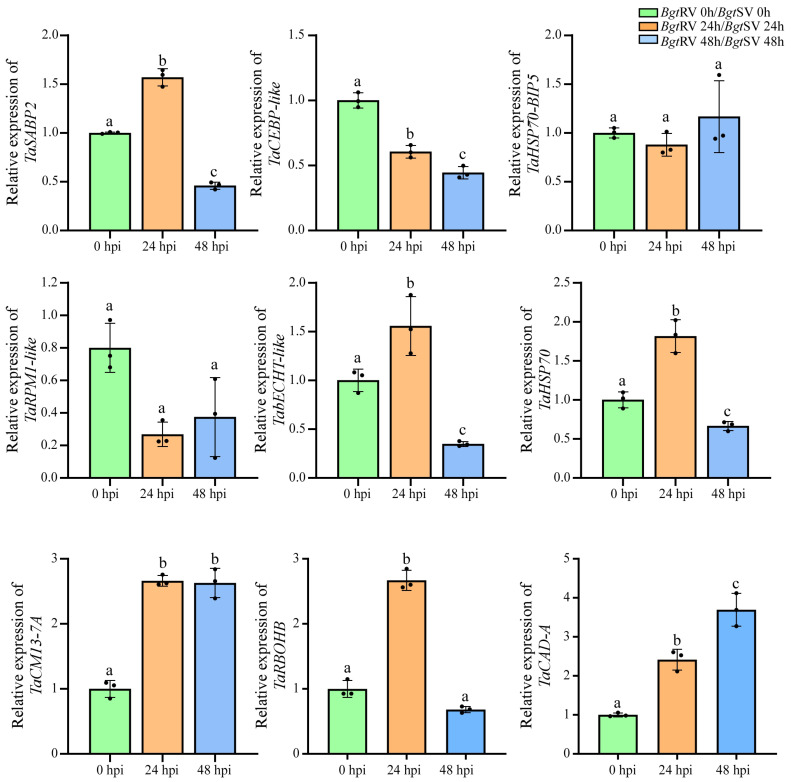
Quantification of twelve gene transcripts at different time points post-inoculation with *Bgt* in wheat. The bar graph shows the fold changes in mRNA expression levels. The green, yellow and blue columns are representatives of inoculation at 0 h, 24 h and 48 h, respectively. The mRNA expression levels were quantified by qRT-PCR normalized to actin. Data represent the mean ± SE from three independent replicates. Significant differences between groups were marked with different letters (a, b, c), and the same letter meant no significant difference (*p* < 0.05). Data are shown as mean ± SD.

To investigate the roles of the proteins involved in the biosynthesis of secondary metabolites and classic immune-related proteins in wheat resistance to *Bgt*, reverse transcription quantitative PCR (RT–qPCR) was performed to verify whether the gene transcript levels were consistent with the proteomic profiles obtained upon *Bgt* infection. We selected 20 genes encoding DEPs in the PPI networks induced by *Bgt*. Transcript levels of 15 genes were successfully quantified, with 9 showing correlation with corresponding protein levels from proteomic data at at least one time point. Furthermore, we found that the expression of *TaCAD-A1* (XM_044546804.1), which encodes cinnamyl alcohol dehydrogenase, was consistent at the mRNA and protein levels with the highest fold change in the resistant wheat (Figure 5). Thus, *TaCAD-A1* was selected for further study.

### 3.6. Silencing of TaCAD-A1 Attenuates Wheat Resistance to Bgt

**Figure 6:**
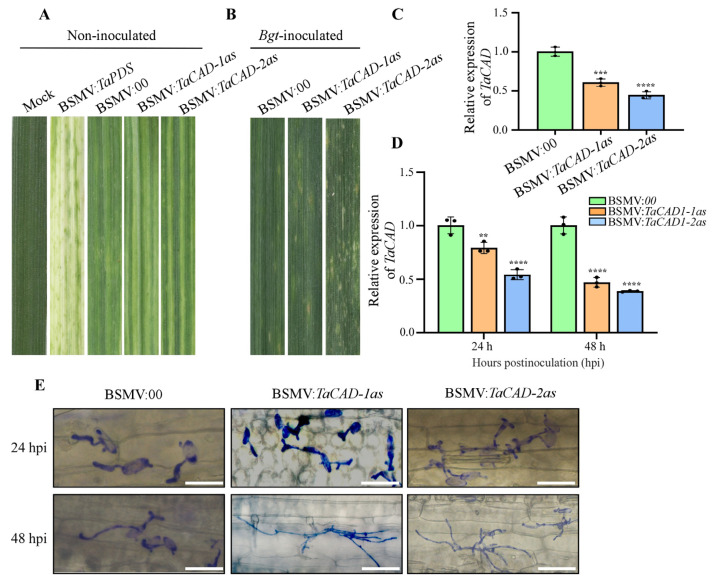
*TaCAD-A1* function analysis by BSMV–VIGS system. (**A**) Phenotypes of wheat leaves inoculated with BSMV:*TaPDS*, FES buffer (mock), BSMV:00, and BSMV:*TaCAD-A1* at 10 dpi. (**B**) Phenotypes of the wheat seedling leaves inoculated with *Bgt* at 10 dpi. Plants were pre-inoculated with BSMV:00 and BSMV:*TaCAD-A1*. (**C**) Relative expression of *TaCAD-A1* in BSMV:00, BSMV:*TaCAD-A1*. (**D**) Relative transcript levels of *TaCAD-A1* in silenced plants inoculated with *Bgt* at 0, 24 and 48 hpi. The mRNA expression levels were quantified by qRT-PCR normalized to actin. Data represent the mean ± SE from three independent replicates. Asterisks denote significant differences at corresponding time points as determined by Student’s *t*-test (** *p* < 0.01, *** *p* < 0.001,**** *p* < 0.0001). (**E**) The growth of *Bgt* in BSMV:00 and BSMV:*TaCAD-A1* wheat leaves was observed via microscopy. Wheat leaves inoculated with *Bgt* were sampled at 24 hpi and 48 hpi. Scale bars = 50 μm.

To investigate the roles of *TaCAD-A1* in the wheat–*Bgt* interaction, we used the barley stripe mosaic virus (BSMV)-mediated virus-induced gene silencing (VIGS) system to silence *TaCAD-A1* in wheat seedling leaves. BSMV:00 (negative control), BSMV:*TaPDS* (positive control), BSMV:*TaCAD-A1* were separately inoculated onto wheat seedling leaves. At 10 days post-vector inoculation, the leaves of the inoculated wheat plants presented typical BSMV infection symptoms on newly emerged leaves, and the BSMV:*TaPDS* plants presented a bleaching phenotype (Figure 6A). The *Bgt* resistance of the plants inoculated with BSMV:*TaCAD-A1* was considerably lower than that of the plants inoculated with BSMV:00 at 10 days post-inoculation (Figure 6B).qRT–PCR analyses revealed that compared with that in the BSMV:00-infected plants, the BSMV:*TaCAD-A1* transcript level was 60% lower (Figure 6C). These data indicated that *TaCAD-A1* was successfully silenced. These plants were further inoculated with *Bgt* to evaluate the role of *TaCAD-A1* in defense. A comparison of the transcript levels between the BSMV:*TaCAD-A1* plants and the control plants revealed that the BSMV:*TaCAD-A1* transcripts were reduced by 50% (Figure 6D), and microscopic observation revealed that *Bgt* progressed much faster in the *TaCAD-A1*-silenced plants than in the control plants at 24 and 48 h post-inoculation with *Bgt* (Figure 6E). These results indicated that the reduced resistance of Yannong37 plants to *Bgt* may be due to the silencing of *TaCAD-A1*. In conclusion, *TaCAD-A1* may participate in wheat defense responses against *Bgt*.

### 3.7. TaCAD-A1 Positively Regulates Defense- and Monolignol Biosynthesis-Related Genes

Defense genes play a crucial role in regulating plant resistance against pathogenic infections. Notably, genes associated with monolignol biosynthesis, including CAD, CRR and COMT, have also been implicated in regulating pathogen resistance. To investigate whether *TaCAD-A1* modulates the expression of defense genes and monolignol biosynthesis-related genes during wheat’s resistance response to *Bgt* infection, we analyzed the transcriptional dynamics of a set of target genes in *TaCAD-A1*-silenced wheat plants and their corresponding control plants. The target genes included four defense-related genes (*Defensin*, *PR10*, *PR17c*, and *chitinase1*) and two monolignol biosynthesis-related genes (*TaCCR* and *TaCOMT1*). Following inoculation with *Bgt* for 24 hpi and 48 hpi, the transcriptional levels of these genes were significantly decreased in *TaCAD-A1*-silenced wheat plants which exhibited reduced resistance to *Bgt* compared to the control plants (infected with BSMV:00).The results suggested that *TaCAD-A1* positively contribute to the expression of defense genes and monolignol biosynthesis-related genes in wheat.

## 4. Discussion

### 4.1. The Abundance of Proteins Enriched in the Cell Wall and Plasmodesmata Was Greater in Resistant Wheat Cultivars

In our study, a total of 7112 proteins were identified in Yannong37 and Yannong1766 during *Bgt* infection at 24 h post-inoculation. Compared with that in the susceptible variety Yannong1766, the protein abundance in the resistant variety significantly increased at 24 h post-inoculation (hpi) (Figure 1E). The cell wall and plasmodesmata are essential for the response to pathogenic invasion [28]. The plant cell wall serves as the primary barrier against pathogen invasion. To reinforce cell walls against fungal pathogens, plants synthesize callose, which interacts with cellulose-forming papillae [29,30]. Callose restricts the spread of microbial infections by impeding the intercellular transport of proteins and effectors via symplastic pathways formed by plasmodesmata [31]. When plants initiate a defensive response, they regulate the levels of callose and the activities of enzymes associated with plasmodesmata. This leads to the synthesis or degradation of callose depending on the type of stress they are experiencing, which in turn adjusts the permeability of plasmodesmata [32]. Feng et al. reported that a viral protein activates the MAPK pathway to promote viral infection by downregulating callose deposition in plants [33]. A recent study revealed that rice stripe virus (RSV) can disrupt the S-acylation of remorin proteins and trigger their autophagic degradation, leading to reduced callose deposition and enhanced viral infection [34]. These findings suggest that pathogens may counteract plant defense responses by affecting callose deposition at plasmodesmata. In our study, compared with those in the susceptible variety, proteins enriched in plasmodesmata and cell wall-related processes showed greater abundance in the resistant variety (Figure 2H). These observations suggest a possible association between powdery mildew resistance and cell wall-related defense responses. However, direct experimental validation, including callose staining and lignin quantification, will be necessary to further verify these potential mechanisms. Despite these findings, several limitations should be considered. In this study, only two time points (0 and 24 hpi) were included in the proteomic analysis, which may not fully capture the dynamic nature of plant defense responses. Future studies incorporating additional time points would help to better resolve the temporal dynamics of wheat responses to *Bgt* infection.

### 4.2. Proteins Related to the Biosynthesis of Secondary Metabolites Are Involved in Bgt Infection

Plants utilize two primary inducible defense mechanisms to defend against pathogen invasion: pathogen-associated molecular pattern (PAMP)- and host damage-associated molecular pattern (DAMP)-triggered immunity (PTI) and nucleotide-binding domains and leucine-rich repeat-containing protein (NLR, also known as NB-LRRs) effector-triggered immunity (ETI) [35]. To date, more than 110 *Pm* resistance genes have been cataloged in wheat, yet only 21 resistance (*R*) genes effective against wheat powdery mildew (*Pm* genes) have been genetically defined, 13 of which encode NLR receptors with N-terminal coiled-coil (CC) domains [36]. NLRs can either directly detect effectors or serve as helpers to initiate signal transduction [37]. Pathogen perception triggers changes downstream of PRRs and NLRs, including mitogen-activated protein kinase (MAPK) activation, the production of reactive oxygen species (ROS), the hypersensitive response (HR), cell death, callose deposition, lignification and salicylic acid (SA) levels [38,39,40]. In our study, proteins enriched in immune effector processes, PCD- and HR-related processes, the MAPK cascade, and SA biosynthetic/metabolic and SA-mediated signaling pathways showed significant abundance changes in both resistant and susceptible varieties following *Bgt* infection (Figure 3E). These proteins may be associated with wheat responses to powdery mildew infection and could potentially function through interconnected regulatory networks (Figure 4C).

Independent of NLR-mediated plant immunity, plant secondary metabolites are adaptations of plants to the environment resulting from long-term interactions between plants and biotic and abiotic factors during the process of evolution. In our study, the results of Kyoto Encyclopedia of Genes and Genomes (KEGG) enrichment analysis revealed that differentially abundant proteins were enriched in the biosynthesis of secondary metabolites (Figure 4A,B), including phytoalexins, lignin, and callose, which are known to be associated with plant defense against pathogen infection [41]. These findings suggest that secondary metabolism-related proteins may be involved in wheat responses to *Bgt* infection. Lignification has the potential to act in several ways in plant against pathogen infection. OsCPS4, which is involved in the biosynthesis of phytoalexins and oryzalexin S, improved rice blast resistance [42]. Li et al. reported that OsCyp71Z2 finely regulates phytoalexin biosynthesis in rice and that *OsCyp71Z2* over-expression in wild-type rice confers resistance to *M. oryzae* [43]. However, these interactions were predicted based on bioinformatic models and therefore should be interpreted with caution. Further experimental validation of key interactions will be necessary to confirm the proposed regulatory relationships.

### 4.3. Protein TaCAD-A1 Enhances Wheat Resistance to Bgt by Reprogramming Defense Genes

**Figure 7:**
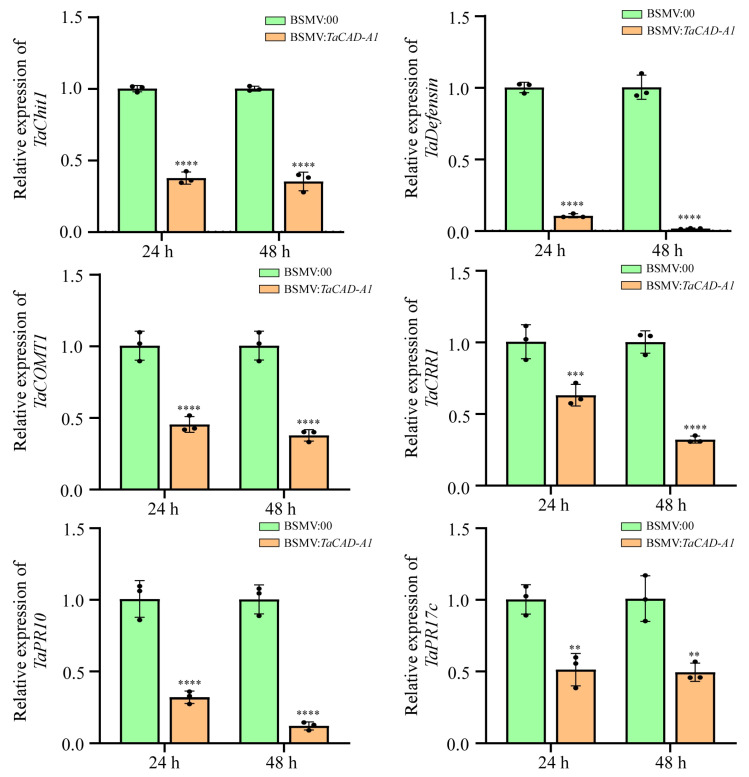
The transcriptional levels of defense and monolignol biosynthesis-related genes after *Bgt* inoculation at 24 h and 48 h. The bar graph shows the fold changes in mRNA expression levels. The green and yellow columns are representatives of inoculation at 24 h and 48 h, respectively. The mRNA expression levels were quantified by qRT-PCR normalized to actin. Data represent the mean ± SE from three independent replicates. Asterisks denote significant differences at corresponding time points as determined by Student’s *t*-test (** *p* < 0.01, *** *p* < 0.001, **** *p* < 0.0001).

In response to fungal infections, plants undergo important processes such as cell wall reinforcement and lignification. Lignin, which is a major component of secondary cell walls, is essential for host defense against pathogens [44]. The synthesis of lignin is a detailed process that requires more than 11 enzymes with three main branches. Cinnamyl alcohol dehydrogenase (CAD) catalyzes the final reduction of cinnamaldehydes to their corresponding alcohols [45]. Previous studies have shown that CAD family genes may contribute to plant defense mechanisms against biotic stresses via the biosynthesis of lignin and/or lignans. Comprehensive transcriptomic analyses revealed *TaCAD* genes contribute to non-redundant functions during wheat growth and development and provide tolerance to diverse abiotic and biotic stresses [46]. Rong et al. reported that *TaCAD12* positively contributes to resistance against sharp eyespot through the regulation of the expression of certain defense genes and monolignol biosynthesis-related genes in wheat [47]. A study on the major genes involved in lignin biosynthesis in *Arabidopsis thaliana* revealed that CAD-C and CAD-D are key components in the defense against both virulent and avirulent strains of the bacterial pathogen *Pseudomonas syringae* pv. tomato, possibly through the salicylic acid defense pathway [48]. A study on soybean mosaic virus (SMV) revealed that GsCAD1 enhances resistance to SMV in soybeans, most likely by affecting the contents of lignin and SA [49]. These findings indicate that CAD can participate in plant defense against fungal, bacterial, and viral infections by catalyzing the synthesis of monolignols or regulating the synthesis of salicylic acid (SA). In our study, BSMV-VIGS demonstrated that *TaCAD-A1* is critical for the gain of function of powdery mildew disease resistance (Figure 6E,F). Although TaCAD-A1 was functionally analyzed, its precise role in wheat defense remains unclear. In plants, defense genes play important roles in resistance to pathogens. The transcriptional levels of four wheat defense marker genes were exhibited in *TaCAD-A1*-silencing wheat plants (Figure 7). These data suggested that *TaCAD-A1* modulates defense-related genes leading to increased wheat resistance response to *Bgt*. Further studies are needed to determine whether it directly participates in lignin biosynthesis, defense signaling pathways, or broader regulatory networks.

## 5. Conclusions

In conclusion, TMT-based proteomic analysis revealed substantial changes in protein abundance in the resistant wheat cultivar Yannong37 and the susceptible cultivar Yannong1766 following *Bgt* infection. Compared with the susceptible cultivar, resistant wheat showed increased abundance of proteins associated with the cell wall and plasmodesmata after powdery mildew infection. In addition, the abundance of several classical disease resistance-related proteins and secondary metabolism-related proteins differed significantly in resistant wheat following *Bgt* infection, and these proteins may participate in complex interaction networks. Furthermore, lignin biosynthesis-related proteins accumulated in resistant wheat after *Bgt* infection, suggesting a potential role in resistance to the pathogen.

## Data Availability

The original contributions presented in this study are available on request from the authors. Further inquiries can be directed to the corresponding authors.

## Supplementary Materials

The following supporting information can be downloaded at https://www.mdpi.com/article/10.3390/life16060872/s1, Table S1: Overview of *Bgt*-infected wheat protein abundance in Yannong37 and Yannong1766; Table S2: Yannong37 and Yannong1766 common differentially abundant proteins in various organelles post-*Bgt* infection; Table S3: Yannong37 specific differentially abundant proteins post-*Bgt* infection; Table S4: Yannong37 differentially abundant proteins associated with the classical disease resistance pathway; Table S5: Gene-specific primers.

## Abbreviations

The following abbreviations are used in this manuscript:

*Bgt*	*Blumeria graminis* f. sp. *tritici*
*Bgt*RV	*Bgt*-resistant variety
*Bgt*SV	*Bgt*-susceptible variety
PGT	primary germ tube
AGT	appressorial germ tube
APP	hooked apical appressorium
SH	secondary hyphae
DEPs	differential expressed proteins
KEGG	Kyoto Encyclopedia of Genes and Genomes
GO	Gene Ontology
BP	biological process
CC	cellular component
PPI	protein–protein interaction
BSMV-VIGS	Barley stripe mosaic virus-induced gene silencing
